# Supplementary material for: Expanding the drug discovery space with predicted metabolite–target interactions
Source: Commun Biol. 2021 Mar 5;4:288. doi: 10.1038/s42003-021-01822-x (PMC7935942; doi:10.1038/s42003-021-01822-x)
Supplement: Supplementary file 2 — Supplementary Information [file 42003_2021_1822_MOESM2_ESM.pdf]

Expanding the drug discovery space with predicted metabolite-target interactions

Supplementary Materials 1

Andrea Nuzzo[1&], Somdutta Saha[1#], Ellen Berg[2], Channa Jayawickreme[1], Joel Tocker[2], James R. Brown[1*%]

[1] GlaxoSmithKline Pharma R&D, Collegeville, Pennsylvania, USA

[2] Eurofins Discovery, Burlingame CA, USA

[*] Primary corresponding author: James.R.Brown@gsk.com

[&]Secondary corresponding author: andrea.8.nuzzo@gsk.com

[#]Current address: EMD Serono Research & Development Institute, Inc., 45A Middlesex Turnpike, Billerica, MA 01821 USA

[%]Current address: Kaleido Biosciences, Inc. 65 Hayden Avenue, Lexington, MA 02421 USA

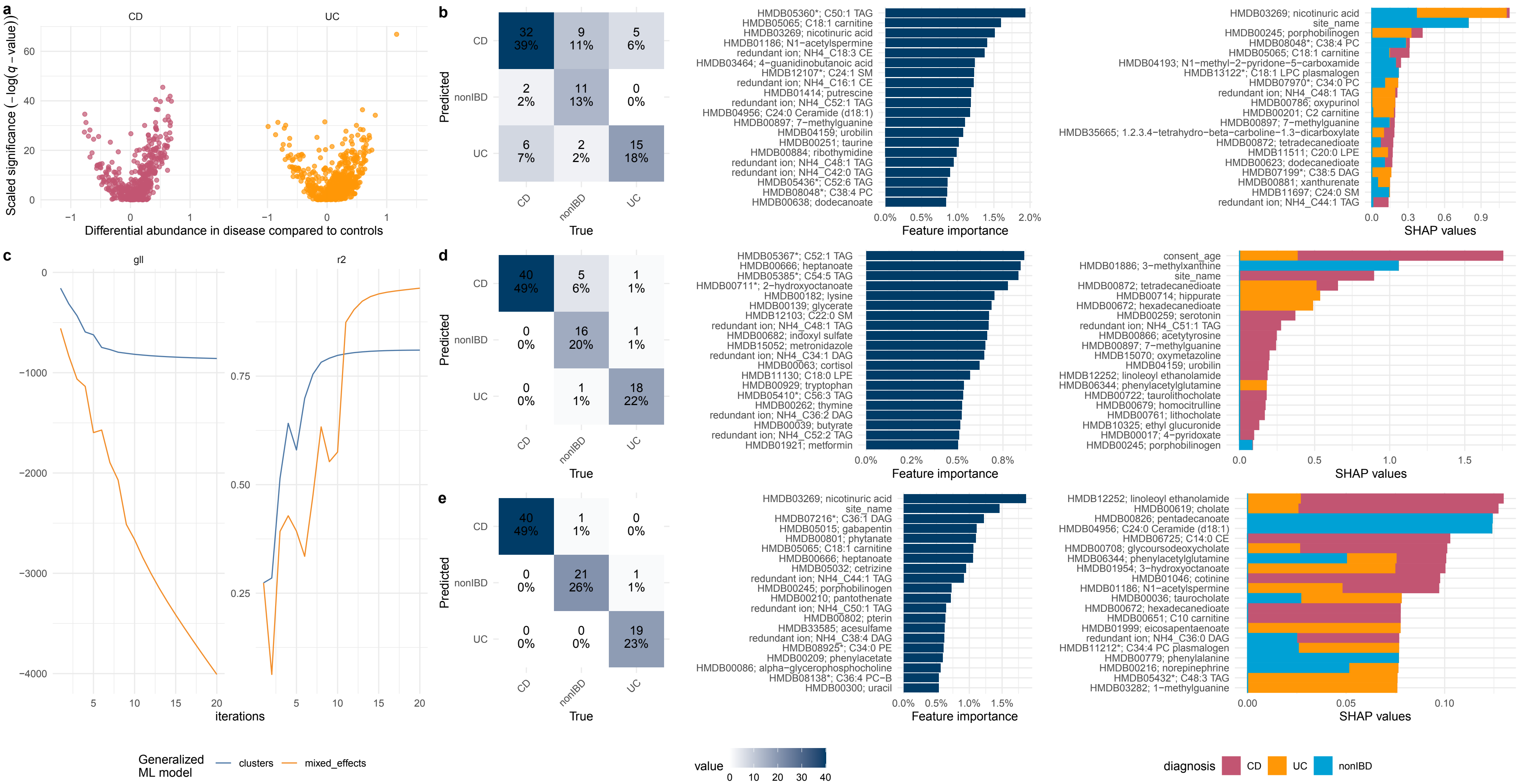

Supplementary Fig. 1. Metabolomics results from each analytical method. (a) Volcano plots depicting the differential abundance per each nmetabolite in Crohn's disease (CD) and Ulcerative Colitis (UC) patients by FDRadjusted p-value using bootstrapped power estimation and Mann-WitheyU test. (b) Confusion matrix, top 20 feature importance and top 20 SHAP values per features obtained from the XGBoost classifier analysis. (c) Generalized log-likelihood (gll) and r-squared (r2) for the generalized XGBoost classifier based on cluster only (blue) and clusters + random effects (orange). (d) Confusion matrix, top 20 feature importance and top 20 SHAP values per features obtained from the generalized XGBoost classifier based on cluster only. (e) Confusion matrix, top 20 feature importance and top 20 SHAP values per features obtained from the generalized XGBoost classifier based on clusters + random effects.

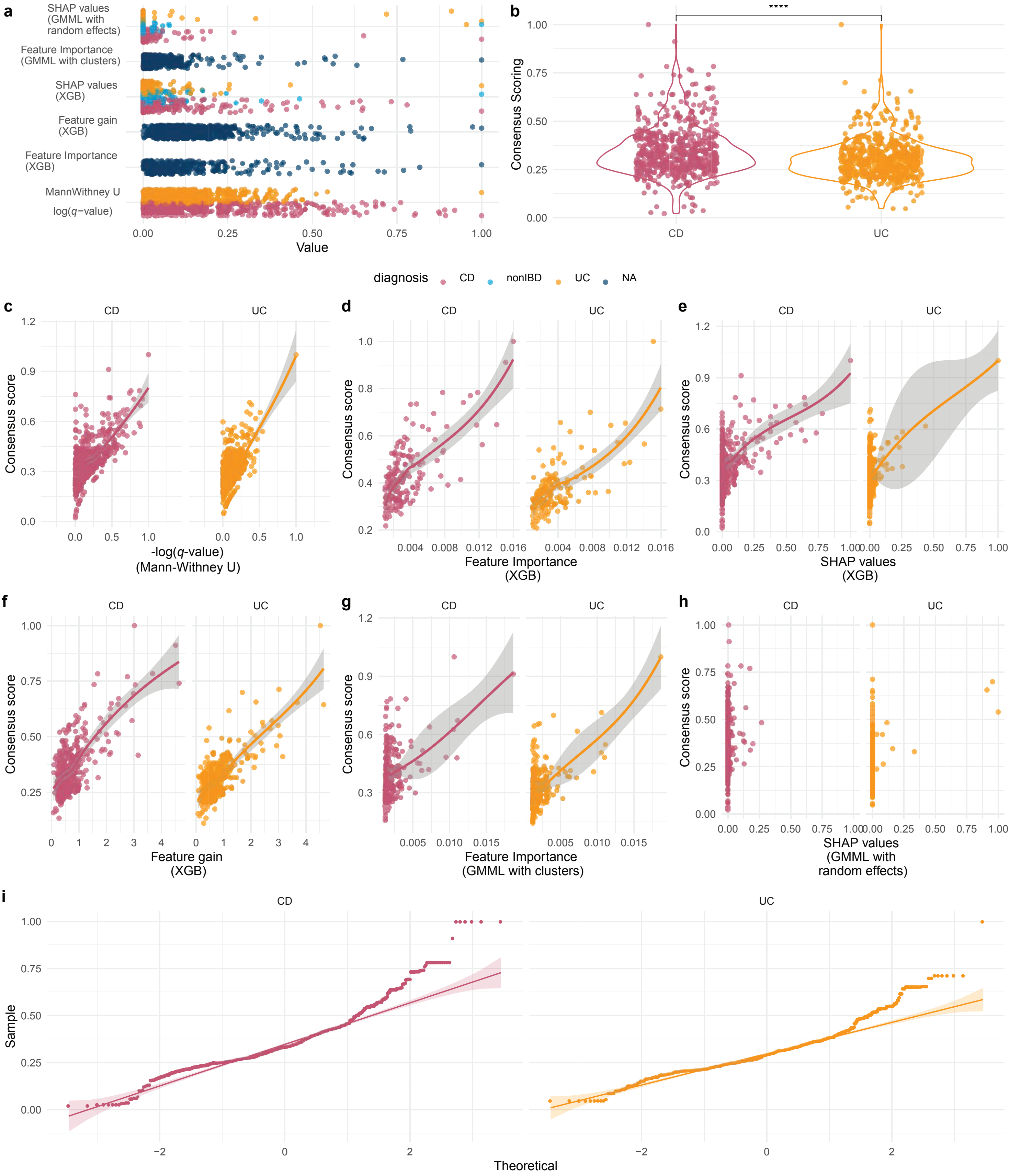

Supplementary Fig. 2. Consensus score diagnostic plot. (a) Distribution of the single descriptors selected from each model, divided by disease state if the method recognizes the category before computation of the consensus scoring. (b) Distribution of the computed consensus score per each metabolite in Crohn's disease (CD) and Ulcerative Colitis (UC) patients. (c-h) Correlation plot between each single indicator used to discriminate disease classes (horizontal axes) and consensus scoring (vertical axes). (i) The qqplot of consensus scoring per disease state.

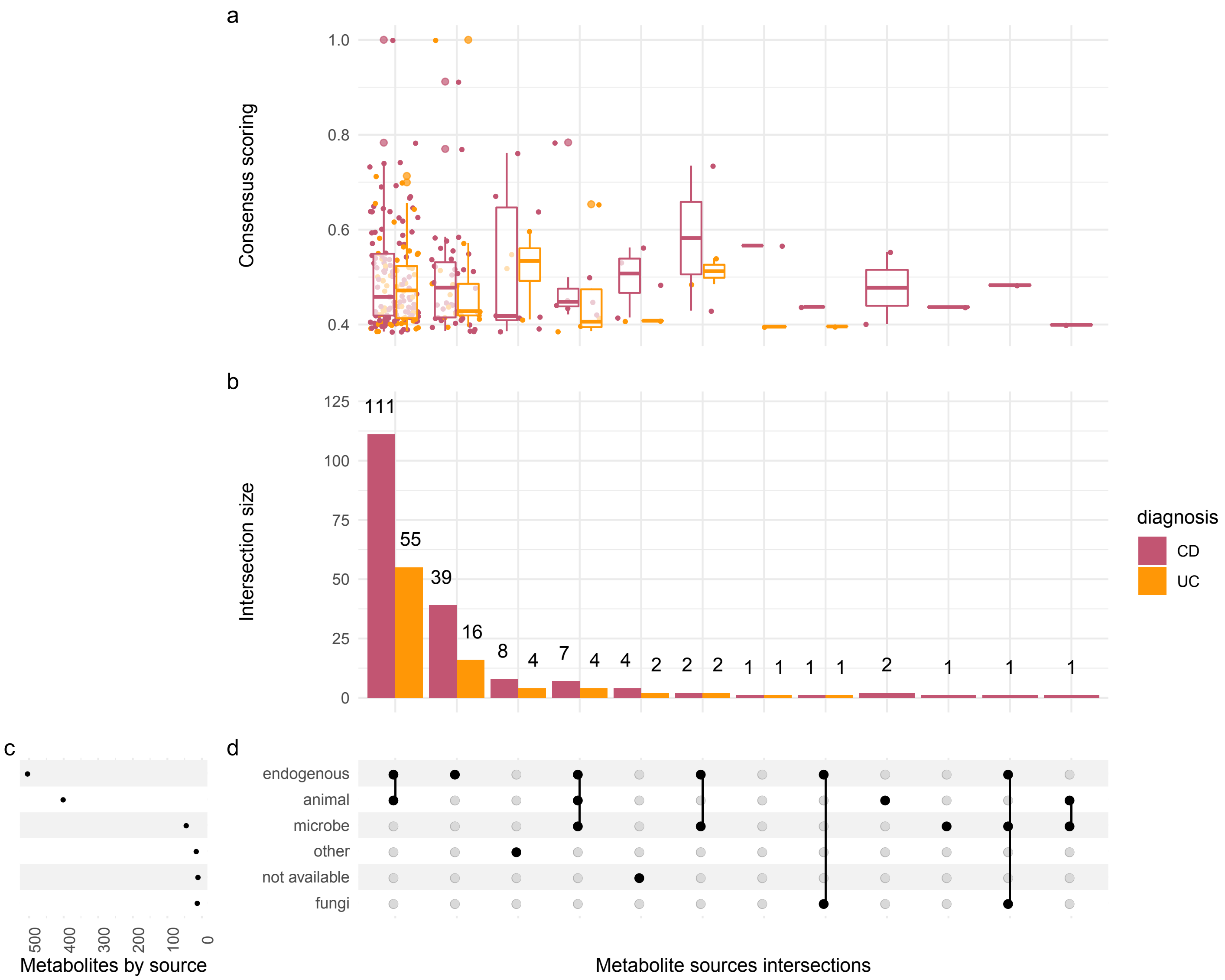

Supplementary Fig. 3. Ontological analysis of the selected 192 metabolites. (a) distribution of the consensus scores of all metabolites by group of origin. Wilcoxon ranked test was used to compare between CD and UC for each group, no significant difference was detected. Hinges represent first and third quartiles, while whiskers represent 95% CI. (b) Number of metabolites ranking in the top quartile of the consensus scoring per each origin subgroup. (c) total number of metabolites per each origin. (d) intersection matrix describing the overlapping origins of metabolites.

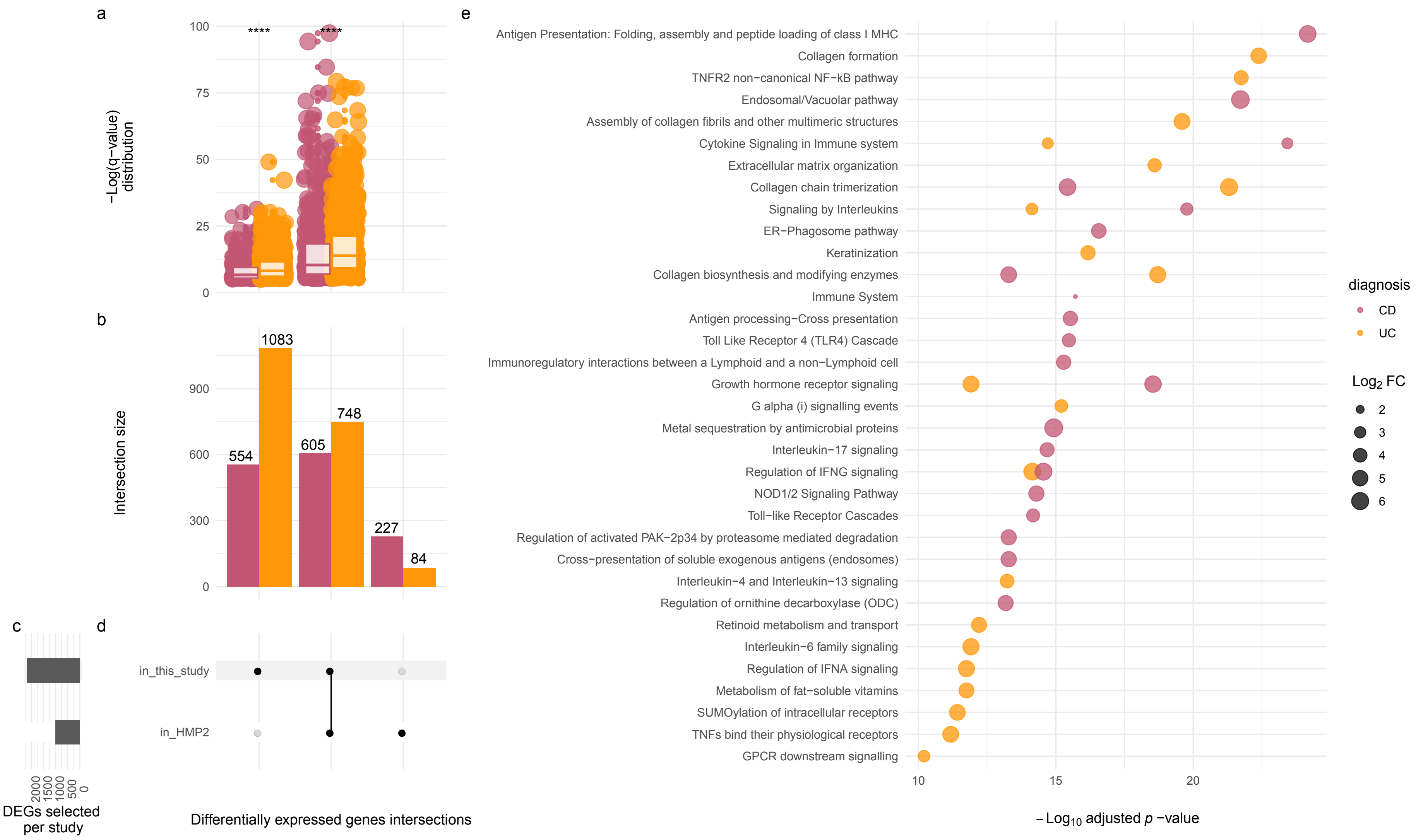

Supplementary Fig. 4. Overview of transcriptomics results. (a) Distribution of the Benjamini-Hochberg FDR-adjusted p-values for each differentially abundant gene selected in the present study subdivided by disease state and overlapping subsets with the original IBD HMP2 study (HMP2). Hinges represent first and third quartiles, while whiskers represent 95% CI. (b) Number of differentially expressed targets selected in this study subdivided per disease states and overlapping subsets with HMP2. (c) Total number of selected differentially expressed genes for each study. (d) Intersection matrix of the differentially expressed genes between the two studies. (e) Pathway enrichment analysis of the differentially expressed genes selected in this study using protein-protein interaction against the REAC-TOME database.

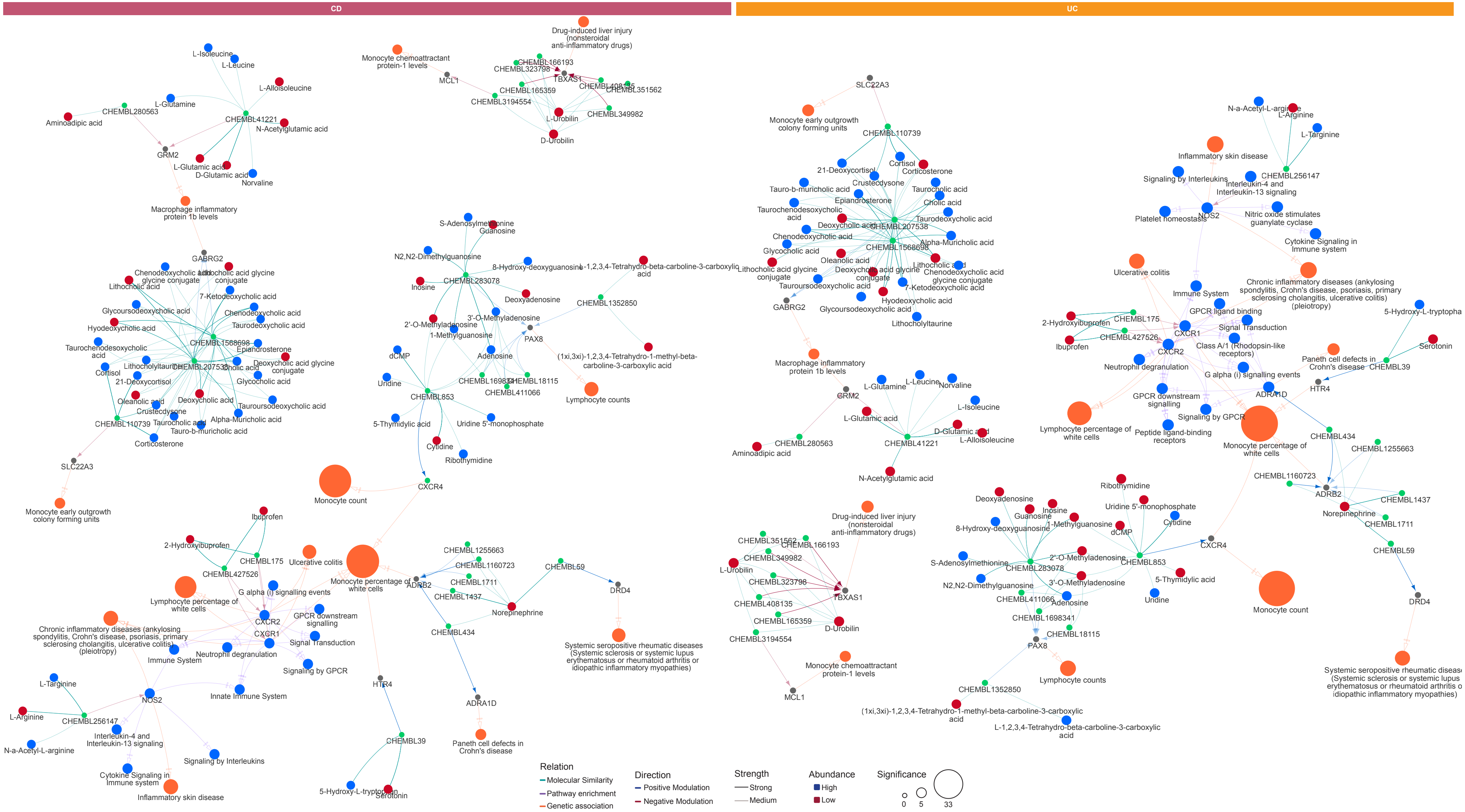

Supplementary Fig. 5. Overview of the connections discovered for the targets. Metabolites were connected through similar ChEMBL compounds where similarity is classified as good (0.8 ≤ Tanimoto score < 0.9) or strong (Tanimoto score ≥ 0.9). Direction and affinity of analog binding to target was parsed from ChEMBL assay databases and it's represented as good (5.5 ≤ pxC50 < 7) and strong (pxC50 ≥ 7). Direction (i.e. up- or down-) of differential expression for targets or differential abundance for metabolites are represented by colors. Significance expresses -log(q-value) of significance tests for either targets differential expression or metabolites differential abundance.

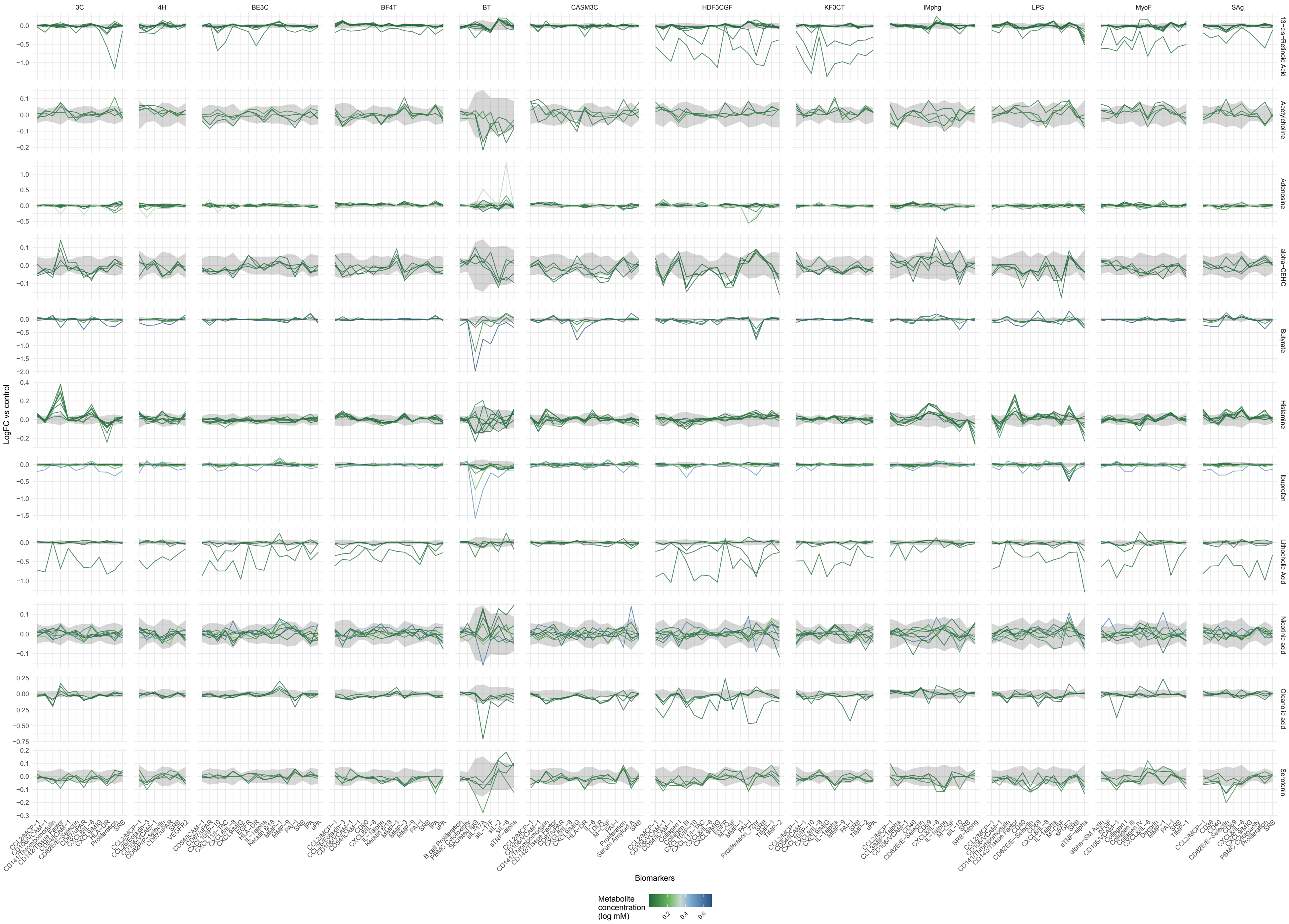

Supplementary Fig. 6. BioMAP profiling results of the in vitro cell tests. Lines represent log-fold change of selected biomarker readouts vs vehicle control per each cell type/system. Due to the different concentration chosen for each metabolite, concentration levels are ranked from lower to higher. Grey areas denote confidence intervals at 95%. Methods section has full descriptions of assay acronyms.
