# Supplementary material for: Expanding the drug discovery space with predicted metabolite–target interactions
Source: Commun Biol. 2021 Mar 5;4:288. doi: 10.1038/s42003-021-01822-x (PMC7935942; doi:10.1038/s42003-021-01822-x)
Supplement: Supplementary file 3 — Description of Additional Supplementary Files [file 42003_2021_1822_MOESM3_ESM.pdf]

Description of Additional Supplementary Files

File Name: Supplementary Data 1

Description: Metabolomics results per each metabolite per disease state. bPE=bootstrapped power estimation, MWU= Mann-WitheyU test, XGB=XGBoost classifier, gXGB = generalized XGBoost classifier with random effects.

File Name: Supplementary Data 2

Description: Candidate metabolites with immunomodulating activities and connected candidate target hits (regardless of the mechanism of action of their analog). Metabolites were selected if ranking at the top quartile and with < 20 target hits (tot 228). Analogs were filtered by Tanimoto > 0.85 or Tversky¬=0.05>0.95, and pxC50 > 5.5. Metabolites tested in vitro in this study (‡) or classified as statistically relevant in the HMP2 paper (†) are marked.

File Name: Supplementary Data 3

Description: Targets parsed from differential expression analysis in UC and CD participants from the host transcriptomics dataset of the HMP2 study and perspective connections with metabolites. Targets were selected for having differential abundance in case/control analysis across biopsy locations and if having <20 modulators with high affinity (pxC50 > 5.5) and high similarity to top-quartile ranking metabolites (Tanimoto > 0.85 or Tversky¬=0.05>0.95) (tot 19). Metabolites are marked as: positive modulator or negative modulators depending on their values in the assay databases and scarce or abundant depending on their differential abundance in the disease state. Targets that were classified as DEGs in the original HMP2 study also marked (†) and enriched pathways from protein interaction networks are reported.

File Name: Supplementary Data 4

Description: Targets parsed from GWAS catalog or OMIM databases and associated with IBD phenotypes or from Graham & Xavier (2020) review23. Targets were selected for having differential abundance in case/control analysis across biopsy locations and if having <20 modulators with high affinity (pxC50 > 5.5) and high similarity to top-quartile ranking metabolites (Tanimoto > 0.85 or Tversky¬=0.05>0.95) (tot 6). Metabolites are marked as: positive modulator or negative modulators depending on their values in the assay databases and scarce or abundant depending on their differential abundance in the disease state. Targets that were

classified as DEGs this study also marked (†) and enriched pathways from protein interaction networks are reported.

File Name: Supplementary Data 5

Description: Description of selected targets, shown in Fig. 4, with genetic association to inflammatory bowel disease (IBD) or other inflammatory phenotypes (from GWAS catalog) with connections to candidate modulator metabolites in IBD patients. Variant types are intron variant (IV), regulatory region variant (RRV), missense variant (MSV), intergenic variant (IGV), 3 prime UTR variant (3UTRV) and 5 prime UTR variant (5UTRV). Differentially expressed genes (DEG) up or down regulated in Crohn’s Disease (CD) and/or Ulcerous Colitis (UC) are indicated. For putative metabolite ligands associated with each target protein the disease, inferred directionality and abundances are shown (* indicates consensus scoring lower than threshold). Further information on additional targets is given in Supplementary Data 4.

File Name: Supplementary Data 6

Description: Perspective connections between metabolites and targets where the direction of modulation is known. Analogs were filtered by Tanimoto > 0.85 or Tversky=0.05>0.95, and pxC50 > 5.5. No filtering was done on metabolites, targets or pleiotropy of either (total 296 metabolites and 118 targets).

File Name: Supplementary Data 7

Description: Full table of perspective connections between targets and metabolites. Analogs were filtered by Tanimoto > 0.85 or Tversky=0.05>0.95, and pxC50 > 5.5. No filtering was done on metabolites, targets or pleiotropy of either (toalt 432 metabolites and 152 targets).

File Name: Supplementary Data 8

Description: BIOMap results of the in vitro cell tests. Log-fold change of biomarker readouts are against baseline per each cell type/system. Cell types are : vascular biology model for inflammatory environment Th1-specific (3C) and a Th2-specific (4H); Th1 inflammatory state specific to arterial smooth muscle cells (CASM3C); monocyte-driven Th1 inflammation (LPS); T cell stimulation (SAg); chronic Th1 inflammation driven by macrophage activation (lMphg); T cell-dependent activation of B cells that occurs in germinal centers (BT); Th1-specific (BE3C) and Th2-specific (BF4T) airway inflammation of the lung; myofibroblast-lung tissue remodeling (MyoF); skin biology (KF3CT) and wound healing (HDF3CGF).
